# Pinocembrin Decreases Ventricular Fibrillation Susceptibility in a Rat Model of Depression

**DOI:** 10.3389/fphar.2020.547966

**Published:** 2020-11-24

**Authors:** Tianxin Ye, Cui Zhang, Gang Wu, Weiguo Wan, Yan Guo, Yuhong Fo, Xiuhuan Chen, Xin Liu, Qian Ran, Jinjun Liang, Shaobo Shi, Bo Yang

**Affiliations:** ^1^ Department of Cardiology, Renmin Hospital of Wuhan University, Wuhan, China; ^2^ Cardiovascular Research Institute, Wuhan University, Wuhan, China; ^3^ Hubei Key Laboratory of Cardiology, Wuhan, China

**Keywords:** depression, ventricular fibrillation, ventricular fibrosis, pinocembrin, autonomic remodeling, ventricular electrical remodeling

## Abstract

**Background:** Depression is associated with the increased risk of mortality and morbidity and is an independent risk factor for many cardiovascular diseases. Depression may promote cardiac arrhythmias, but little is known about the mechanisms. Pinocembrin mitigated depressive-like behaviors and exhibited cardioprotective effects in several models; however, whether pinocembrin benefits ventricular arrhythmias in depression models has not been elucidated. Thus, this study was to evaluate the effects of pinocembrin on ventricular fibrillation susceptibility in rat models of depression.

**Methods:** Male Sprague-Dawley rats were randomly assigned into control, control + pinocembrin, MDD (major depressive disorder), and MDP (MDD + pinocembrin) groups, respectively. Depressive-like behaviors, ventricular electrophysiological parameters, electrocardiogram parameters, heart rate variability, ventricular histology, serum norepinephrine, tumor necrosis factor-α, and interleukin-1β were detected. Protein levels in left ventricle were measured by Western blot assays.

**Results:** Compared with the MDD group, pinocembrin significantly mitigated depressive-like behaviors, prolonged ventricular effective refractory period, action potential duration, QT, and corrected QT (QTc) interval, improved heart rate variability, decreased Tpeak–Tend interval, ventricular fibrillation inducibility rate, ventricular fibrosis, ventricular positive nerve densities, and protein expression of tyrosine hydroxylase and growth associated protein-43, reduced serum norepinephrine, tumor necrosis factor-α, interleukin-1β concentrations, and the expression levels of p-IκBα and p-p65, and increased the protein expression of Cx43, Cav1.2, and Kv.4.2 in the MDP group.

**Conclusion:** Pinocembrin attenuates ventricular electrical remodeling, autonomic remodeling, and ion-channel remodeling, lowers ventricular fibrosis, and suppresses depression-induced inflammatory responses, providing new insights in pinocembrin and ventricular arrhythmias in depressed patients.

## Introduction

Sudden cardiac death (SCD), defined as unexpected deaths within a short period after the onset of symptoms, is an enormous public health problem mainly caused by malignant ventricular arrhythmias (VAs) (Al-Khatib et al., 2016; Chatterjee & Albert, 2019). Major depressive disorder (MDD), also simply described as depression, is a serious mental illness with a high lifetime incidence (∼15%) and is projected to be the second most prevalent disease and the leading cause of disability by 2030 (Vavakova et al., 2015; Fabbri, 2016; Hasin et al., 2018). Cardiovascular diseases and depression often coexist and facilitate each other, contributing to a cycle of poor physical and mental health (Lippi et al., 2009; Whooley & Wong, 2013). Previous study indicated that VAs vulnerability was increased in a rat model of depression (Grippo et al., 2004; Liu et al., 2017). Moreover, a clinical study also demonstrated the significant role of depression in developing SCD (Luukinen et al., 2003); however, the underlying mechanisms remain unknown.

Pinocembrin (5,7-dihydroxyflavanone), the most abundant flavonoid in propolis, possesses a variety of pharmacological actions, including the anti-inflammatory, antioxidant, and antiapoptotic effects (Shen et al., 2019). Pinocembrin could attenuate cognitive deficits and inhibit neuronal degeneration in mice models of Alzheimer’s disease (Liu et al., 2014) and improve spatial learning and memory in mice models of diabetes (Pei & Sun, 2018). In addition, pinocembrin decreased cerebral infarct size, improved behavior deficits, and ameliorated neuronal impairment in rats with global cerebral ischemia/reperfusion (I/R) (Shi et al., 2011; Tao et al., 2018). A recent study indicated that pinocembrin improved cardiac function, reduced VAs, and decreased myocardial infarct size in a rat model of myocardial I/R (Lungkaphin et al., 2015). Pinocembrin could also regulate the ion channels (e.g. Na^+^-K^+^ ATPase, Ca^2+^-Mg^2+^ ATPase, and Kir2.1), as well as the gap junction [e.g., Connexin (Cx) 43 Cx43] in myocardial tissues (Zhang et al., 2018). Moreover, our latest study demonstrated that pinocembrin could attenuate autonomic dysfunction and cardiac arrhythmias in myocardial infarction (MI) rats, confirming the cardioprotection of pinocembrin (Ye et al., 2019b). The aforementioned studies demonstrate the neuroprotective and cardioprotective effects of pinocembrin; however, little is known whether pinocembrin attenuates behavior deficits and VF susceptibility in models of depression.

Stressful life events are proposed to be linked to depression and are used to predict the severity of depression (Hammen et al., 1992; Sapolsky, 1996). Chronic unpredictable mild stress (CUMS) is applied to induce mood disorders (e.g., anhedonia and reduced activity levels). Although it remains controversial whether the CUMS model has a high predictive value in depression, this model has been widely used in experimental studies related to depression and considered as the most effective and reliable method to establish a rodent model of depression (Antoniuk et al., 2019). In this study, CUMS was conducted to evaluate the effects of pinocembrin on VF susceptibility and the potential mechanisms in rat models of depression.

## Materials and Methods

### Animals

**FIGURE 1:**
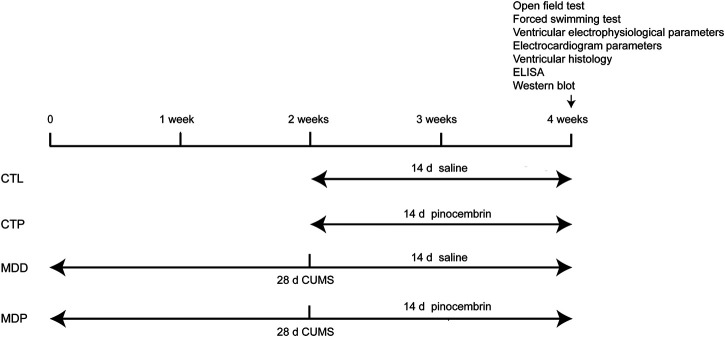
Time axis of procedures used in the present study. CUMS, chronic unpredictable mild stress.

Ninety-six male Sprague-Dawley (200 ± 20 g) rats were randomized into four groups (n = 24 per group): i) CTL group: CTL + saline; ii) CTP group: CTL + pinocembrin (purity ≥ 98%, Sigma–Aldrich); iii) MDD group: 4-week CUMS + saline; and iv) MDP group: 4-week CUMS + pinocembrin. Rats received injection of pinocembrin (5 mg/kg) or saline through the tail vein once a day for 2 weeks (from week 3 to week 4). The dose was chosen according to the previous work (Tao et al., 2018; Ye et al., 2019b) and the duration of dosing was based on the results of preliminary study. Meanwhile, the time axis of procedures in the animals was shown in Figure 1.

Rats were raised in standard conditions (12 h light/dark cycle) and were allowed free access to food and water ad libitum unless otherwise noted. Animal experiments were performed by following the Guide for the Care and Use of Laboratory Animals of US National Institutes of Health after obtaining support from the Animal Ethics Committee of Renmin Hospital of Wuhan University, China. Besides, the ARRIVE Guidelines was implemented in the present study involving rats (Kilkenny et al., 2012).

### Specific Experimental Procedures

#### Chronic Unpredictable Mild Stress

CUMS was carried out to establish rat models of depression as described previously (Liang et al., 2015; Liu et al., 2017). In brief, rats were exposed to one of the following chronic stresses daily for a total of 28 days: 24-h food deprivation, 24-h water deprivation, tail clipping for 1 min, 24-h lighting, 45° cage tilt for 24 h, immobilization for 1 h, forced cold swim at 4°C for 10 min, forced hot swim at 40°C for 10 min, or 24-h cage soiling. Behavioral tests, including the open field test (OFT) and the forced swimming test (FST), were performed to assess depression-like symptoms before and after the 4-week CUMS procedure. Data of behavioral measurements at baseline were displayed in Table 1.

**TABLE 1:** Data of behavioral measurements at baseline in the present study.

Parameters	CTL	CTP	MDD	MDP
OFT parameters				
Traveling distance (m)	31.15 ± 3.84	29.81 ± 3.41	30.25 ± 3.45	30.39 ± 4.06
Traveling speed (cm/s)	9.62 ± 1.38	9.82 ± 1.48	9.56 ± 1.40	9.78 ± 1.42
Rearing	24.96 ± 5.24	25.13 ± 4.74	24.54 ± 4.85	23.88 ± 4.52
FST parameters				
Immobility time (s)	14.79 ± 4.34	16.42 ± 4.74	15.88 ± 4.91	16.17 ± 5.02

Abbreviation: OFT, open field test; FST, forced swimming test.

#### Open Field Test

The OFT was conducted to observe the locomotor activity. In brief, the individual rat was placed into the center of an open field arena (100 × 100 × 40 cm^3^) and was then free to move around for 5 min in each trial, with a video-tracking system (Ethovision 3.0, Noldus) detecting the images and measuring the traveling distance and speed in rats. In addition, the vertical movement (rearing) was also calculated.

#### Forced Swimming Test

The FST was performed to detect the immobility. Briefly, the individual rat was placed in the cylinder (height 50 cm, diameter 20 cm; 40 ± 1.5 cm water at 24 ± 0.5°C) for 6 min in each trial. The rat was allowed to adapt to the new environment in the first 2 min and was then free to move around for 4 min to measure the immobility time.

### Ventricular Electrophysiological Parameters

Rats were anesthetized using sodium pentobarbital [(intraperitoneal injection (i.p.) at a dose of 40 mg/kg; Sigma)] and were heparinized using heparin sodium (400 U; i.p.; Sigma). Hearts were immediately harvested and were perfused according to the Langendorff technique (AD Instruments, Dunedin, New Zealand) as described previously (Liu et al., 2017; Ye et al., 2019a; Ye et al., 2019b). The left ventricular monophasic action potentials (MAPs) were measured via Ag–AgCl electrodes according to previous studies (Shi et al., 2014; Liang et al., 2015; Liu et al., 2017).

#### Effective Refractory Period

The ventricular effective refractory period (ERP) was detected in left ventricle (LV) at the following four epicardial sites: the left anterior base (LAB), left posterior base (LPB), left anterior apex (LAA), and left posterior apex (LPA). A programmed S1S2 stimuli protocol was performed with eight consecutive stimuli (S1) at cycle length (CL) of 250 ms followed by a premature stimulus (S2), with ERP determined as the longest S2 interval that was unable to catch the ventricle (Shi et al., 2014; Liang et al., 2015).

#### Action Potential Duration

The ventricular action potential duration (APD) was detected in LV at aforementioned four epicardial sites. An S1S1 stimulation procedure was conducted with 10 stimuli [pacing cycle length (PCL): 250 ms]. The APD was assessed at 90% repolarization (APD_90_) and 50% repolarization (APD_50_) (Liang et al., 2015; Liu et al., 2017).

#### Ventricular Fibrillation Inducibility

The ventricular fibrillation (VF) inducibility was conducted by a 50 Hz burst pacing lasting for 2 s and repeated no more than 10 times. VF was defined according to the Lambeth Conventions (II) (Curtis et al., 2013) and VF occurring and maintained for at least 2 s was considered the effective VF signal in the present study.

### Electrocardiogram Parameters and Heart Rate Variability

#### Electrocardiogram Parameters

Electrocardiogram (ECG; lead II) was recorded for 15 min under the state of anesthesia. Labchart 8 software was used to analyze the QT interval, corrected QT interval (QTc), Tpeak–Tend interval, and QRS duration. QT interval was corrected followed by the Bazett’s formula: QT/RR^1/2^. Tpeak–Tend interval was detected from the peak to the end of the T wave.

#### Heart Rate Variability

ECG was collected and the RR variability was measured on the segment of 250–300 cycles (Ye et al., 2019a; Ye et al., 2019b). Heart rate variability (HRV) was analyzed in the time domain using LabChart 8.0 software. The time-domain HRV parameters were described in Table 2.

**TABLE 2:** The time-domain HRV parameters in the current study.

Parameters	Description	Autonomic References
Mean HR (bpm)	Mean heart rate	—
Mean RR (ms)	Mean value of all normal RR intervals	—
SDNN (ms)	Standard deviation of normal RR intervals (a measure of overall variability)	Parasympathetic activity
RMSSD (ms)	Square root of the mean squared differences of successive RR intervals, a measure of beat-to-beat variability	Parasympathetic activity

Abbreviation: HRV, heart rate variability; bpm, beat per minute.

### Histological Examination

LV tissues were dissected and fixed in 4% paraformaldehyde for 24 h, embedded in paraffin, and sectioned into 5 µm intervals.

#### Immunohistochemistry

The serial LV sections were incubated with antibodies against tyrosine hydroxylase (TH, 1:500, Cell Signaling Technology, United States) and growth associated protein-43 (GAP43, 1:500, Abcam) for immunohistochemical staining. Image-Pro Plus 6.0 software was applied to analyze the slides (Wang et al., 2019).

#### Masson Staining

The serial LV sections were stained with Masson trichrome method. Image-Pro-Plus 6.0 software was applied to calculate the degree of fibrosis (Liu et al., 2017).

### Enzyme-Linked Immunosorbent Assay

Blood was extracted from the inferior vena cava for ELISA as described in the previous work (Ye et al., 2019b). Serum norepinephrine (NE), TNF-α, and IL-1β were detected. The procedure of ELISA was conducted in accordance with the manufacturers’ specification.

### Western Blot Analysis

LV tissues were obtained and proteins were extracted. Western blot was performed as described in the previous work (Liu et al., 2017). Antibodies included: TH (1:2,000, Cell Signaling Technology, United States), GAP43 (1:1,000; Abcam), Cx43 (1:1,000; Abcam), Cav1.2 (1:500; Abcam), Kv4.2 (1:500; Abcam), IκBα (1:2,000, Cell Signaling Technology, United States), p-IκBα (1:1,000, Cell Signaling Technology, USA), and p-p65 (1:1,000, Cell Signaling Technology, United States). GAPDH (1:1,0000; Abcam) was identified as the internal control.

### Statistical Analysis

Continuous variables were presented as mean ± SEM and proportions were expressed as percentages. Student’s *t*-test or the Pearson chi test was applied for comparison between two groups, and ANOVA followed by post hoc test was used among multiple groups. All tests were 2-tailed, and p < 0.05 was considered to be statistically significant.

## Results

### Pinocembrin Improved Depressive-Like Behaviors

**FIGURE 2:**
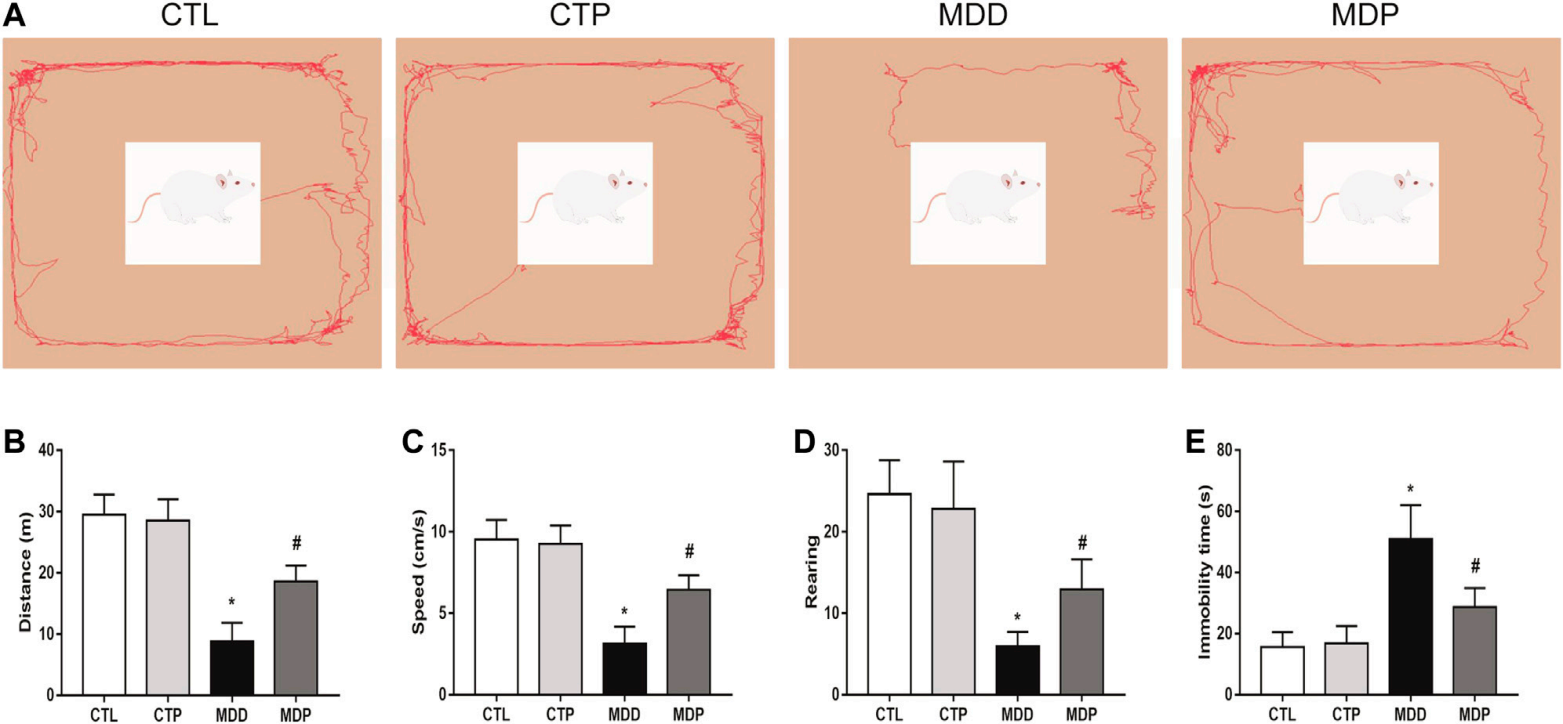
Behavioral tests. **(A)** Representative images of movement locus in OFT. **(B–D)** Traveling distance, speed, and score of rearing in OFT, respectively. n = 24 per group. **(E)** Immobility time in FST. n = 24 per group. *p < 0.01 vs. CTL; #*p* < 0.01 vs. MDD. OFT, open field test; FST, forced swimming test.

After 4-week CUMS, the OFT and FST were conducted to assess the depressive symptoms in rats. In the OFT, Figure 2A showed the representative images of movement locus. Figures 2B–D indicated a significant decrease in the traveling distance, speed, and score of rearing in the MDD group compared with the CTL group. In the FST, a dramatical increase was found in the immobility time in the MDD group compared with the CTL group (50.96 ± 2.26 vs. 15.58 ± 0.99 s, *p* < 0.01; Figure 2E). The aforementioned variables were significantly mitigated in the MDP group compared with the MDD group (Figures 2B–E). In addition, there were no significant changes in the behavioral tests between the CTL group and CTP group.

### Pinocembrin Ameliorated Ventricular Electrical Remodeling

**FIGURE 3:**
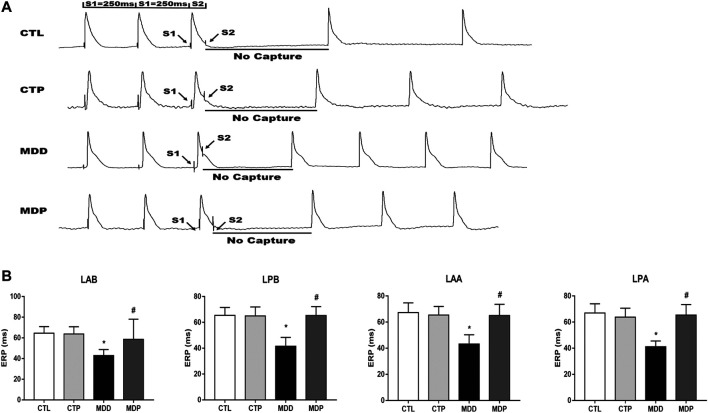
Monophasic action potentials. **(A)** Representative recordings of ventricular electrogram. **(B)** ERP at four epicardial sites, respectively. n = 10 per group. *p < 0.01 vs. CTL; #*p* < 0.01 vs. MDD. ERP, effective refractory period.

**FIGURE 4:**
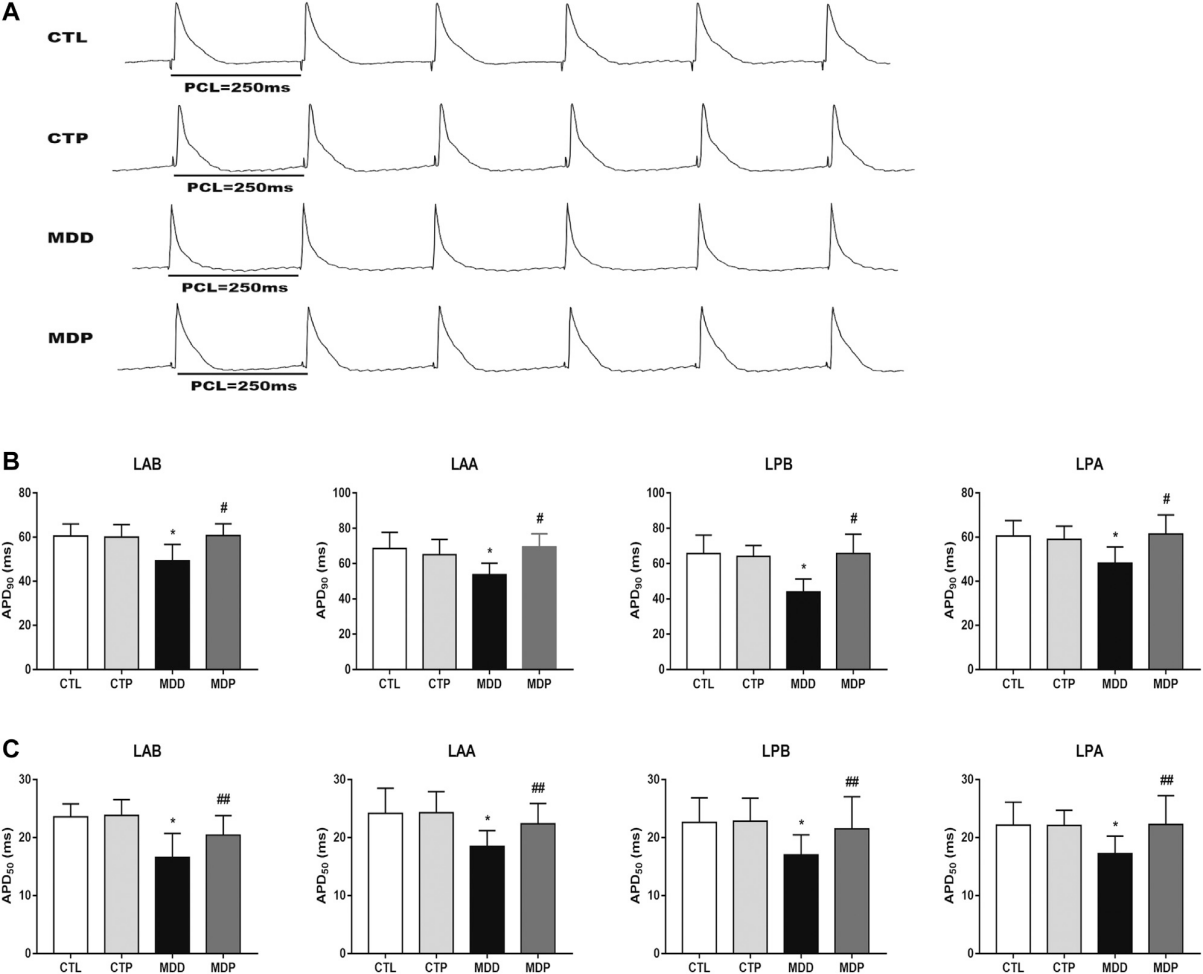
Ventricular repolarization. **(A)** Representative recordings of APD at PCL of 250 ms. **(B,C)** APD_90_ and APD_50_ at four epicardial sites at PCL of 250 ms, respectively. n = 10 per group. *p < 0.01 vs. CTL; #*p* < 0.01 vs. MDD; ##*p* < 0.05 vs. MDD. PCL, pacing cycle length; APD_90_ and APD_50_, action potential duration at 90% repolarization and 50% repolarization, respectively.

**FIGURE 5:**
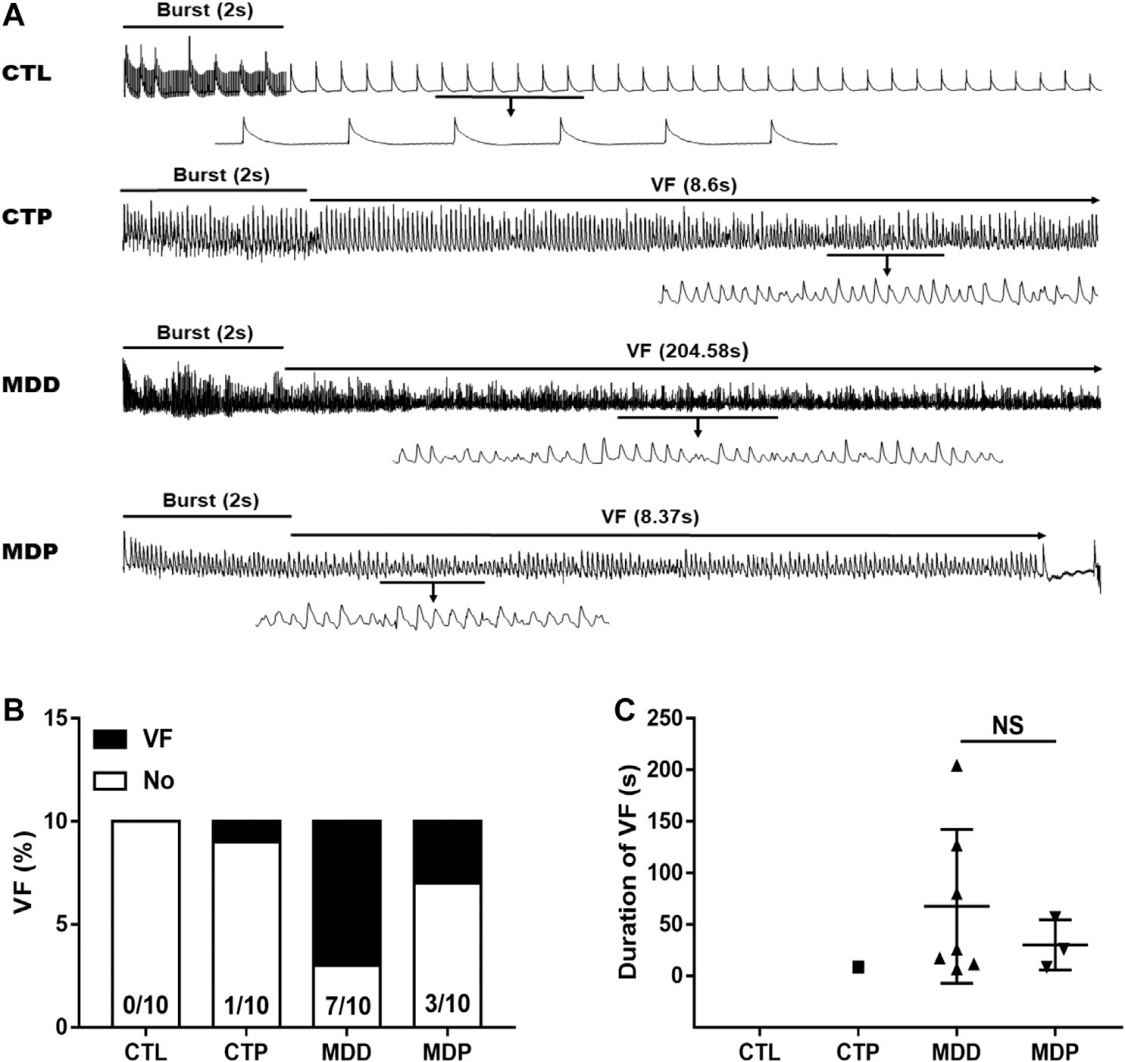
VF vulnerability. **(A)** Representative recordings of burst pacing protocol. **(B,C)** Inducibility and duration of VF, respectively. n = 10 per group. VF, ventricular fibrillation; NS, no significant difference.

Figure 3A displayed the representative recordings of the ventricular electrogram and Figure 4A showed the ventricular APD in the four groups. A significant decrease was found in the mean ERP, APD_90_, and APD_50_ at four epicardial sites in MDD rats compared with the CTL rats, whereas those were significantly prolonged after pinocembrin administration in MDP rats (Figures 3B, 4B,C). Figure 5A showed the representative sample of burst pacing protocol. Although the average VF duration showed no significant difference between the MDD group and MDP group (67.63 ± 28.25 vs. 30.22 ± 14.07 s, *p* > 0.05; Figure 5C), the VF incidence was 1/10 in the CTP group, 7/10 in the MDD group, and 3/10 in the MDP group (Figure 5B). However, no significant differences were observed in ventricular electrophysiological parameters between the CTL group and CTP group.

### Pinocembrin Altered Electrocardiogram Parameters and Improved Heart Rate Variability

**FIGURE 6:**
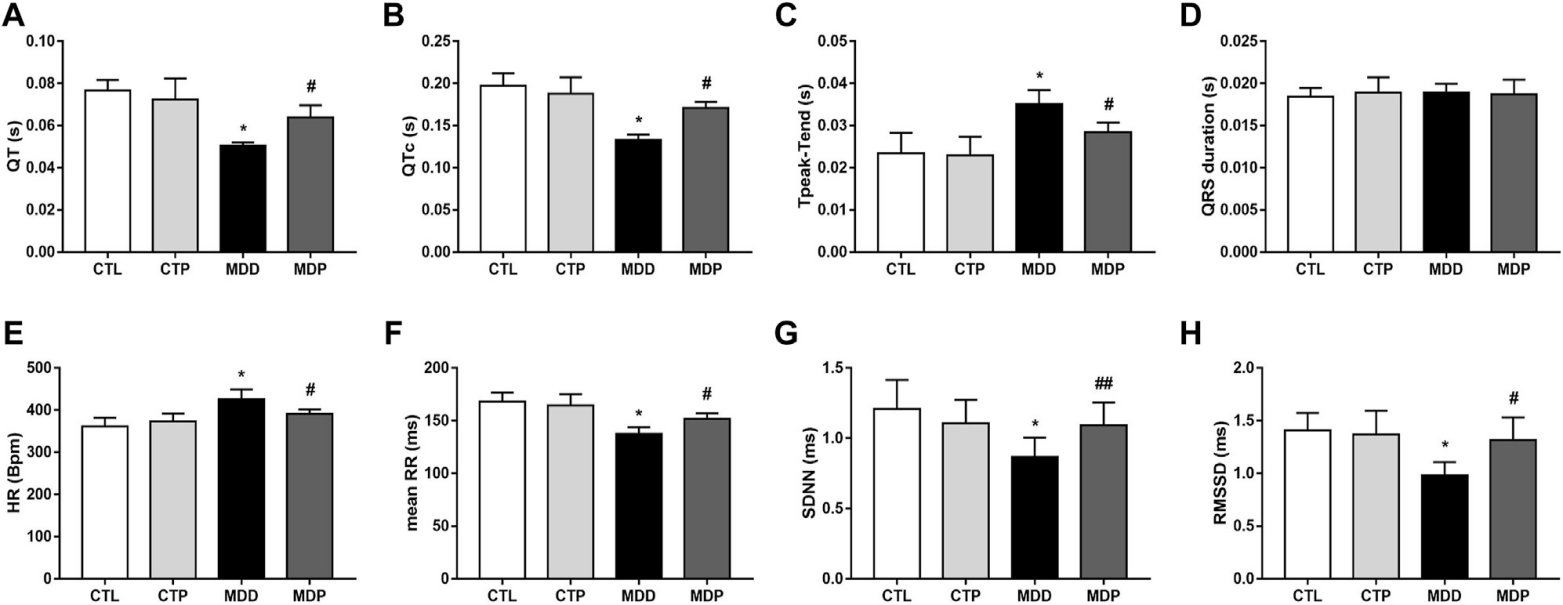
Electrocardiogram parameters and HRV. **(A–D)** QT interval, corrected QT interval, Tpeak–Tend interval and QRS interval, respectively. n = 7 per group. **(E–H)** Statistical analysis of HR, mean RR, SDNN, and RMSSD, respectively. n = 7 per group. *p < 0.01 vs. CTL; #*p* < 0.01 vs. MDD; ##*p* < 0.05 vs. MDD. HRV, heart rate variability.

As Figures 6A–C showed, the QT and QTc interval were significantly decreased, whereas the Tpeak–Tend interval was increased in the MDD group compared with the CTL group. Figure 6D showed no significant difference in the QRS duration within the four groups. In the time-domain HRV variables, compared with the CTL group, the mean heart rate (HR) was significantly increased whereas other variables, including the mean of all normal RR intervals (mean RR), standard deviation of normal RR intervals (SDNN), and square root of the mean squared differences of successive RR intervals (RMSSD), were significantly reduced in the MDD group (Figures 6E–H). The aforementioned alterations were significantly ameliorated after pinocembrin treatment. In addition, the parameters mentioned above showed no significant differences between the CTL group and CTP group.

### Pinocembrin Attenuated Ventricular Autonomic Neural Remodeling

**FIGURE 7:**
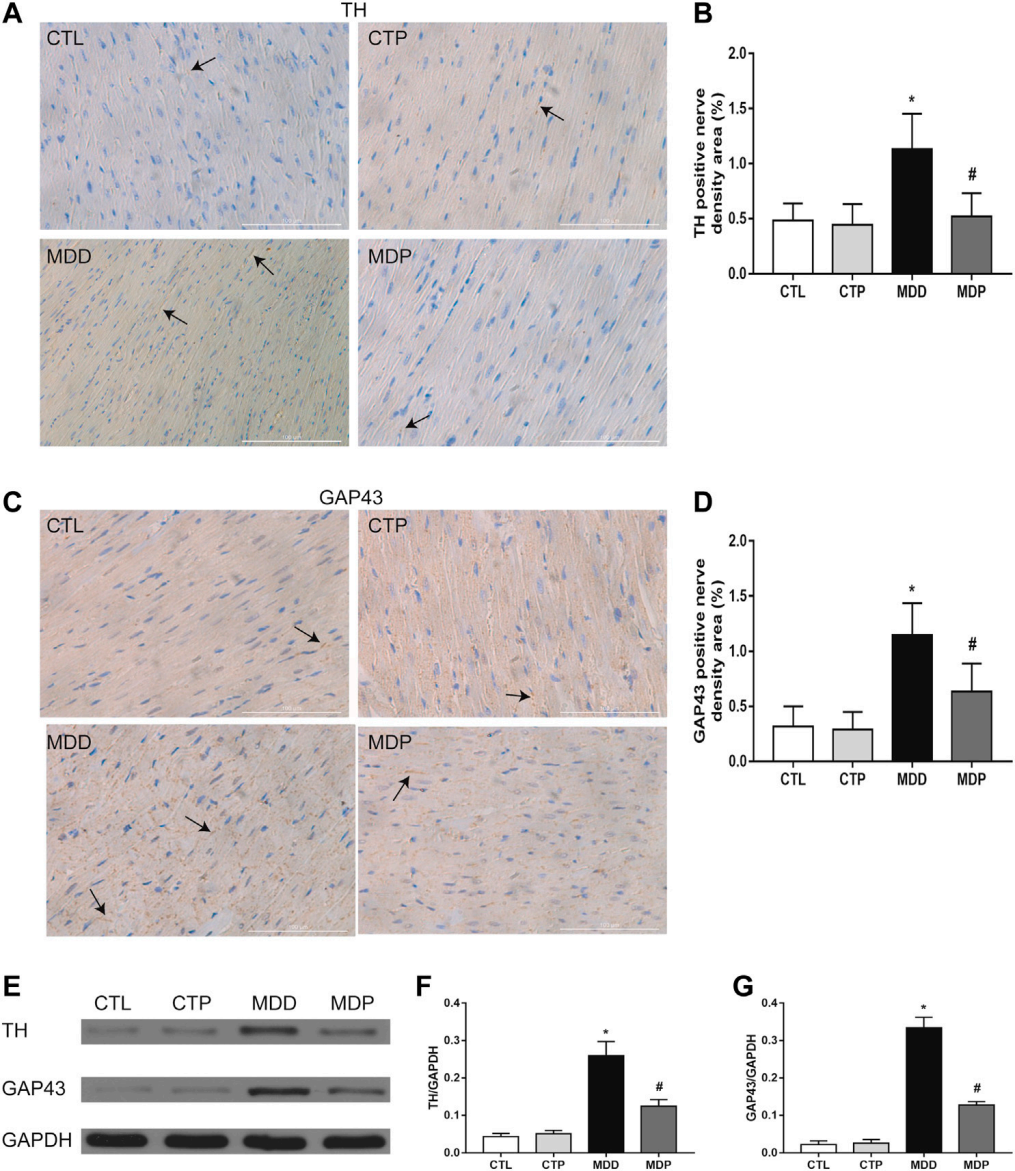
Ventricular autonomic neural remodeling. **(A,B)** Typical staining examples and quantitative analysis of TH in LV, respectively (original magnification: ×400). **(C,D)** Typical staining examples and quantitative analysis of GAP43 in LV, respectively (original magnification: ×400). n = 7 per group. **(E–G)** Immunoblotting and expression ratio of TH and GAP43, respectively. n = 7 per group. *p < 0.01 vs. CTL; #*p* < 0.01 vs. MDD. TH, tyrosine hydroxylase; GAP43, growth associated protein-43; LV, left ventricle.

Figures 7A,C showed the typical sample of TH and GAP43 staining in LV tissues, respectively. Compared with the CTL group, a significant increase was found in both TH and GAP43 positive nerve densities in the MDD group, whereas pinocembrin significantly decreased the positive nerve densities of TH and GAP43 (Figures 7B,D). In addition, the expression of TH and GAP43 in LV tissues was significantly higher in MDD rats than in CTL rats and was then significantly reduced in MDP rats (Figures 7E–G). The staining and expression levels of TH and GAP43 showed no significant differences between the CTL group and CTP group.

### Pinocembrin Reduced Ventricular Fibrosis and NE, TNF-α, and IL-1β, and Increased Cx43 Expression

**FIGURE 8:**
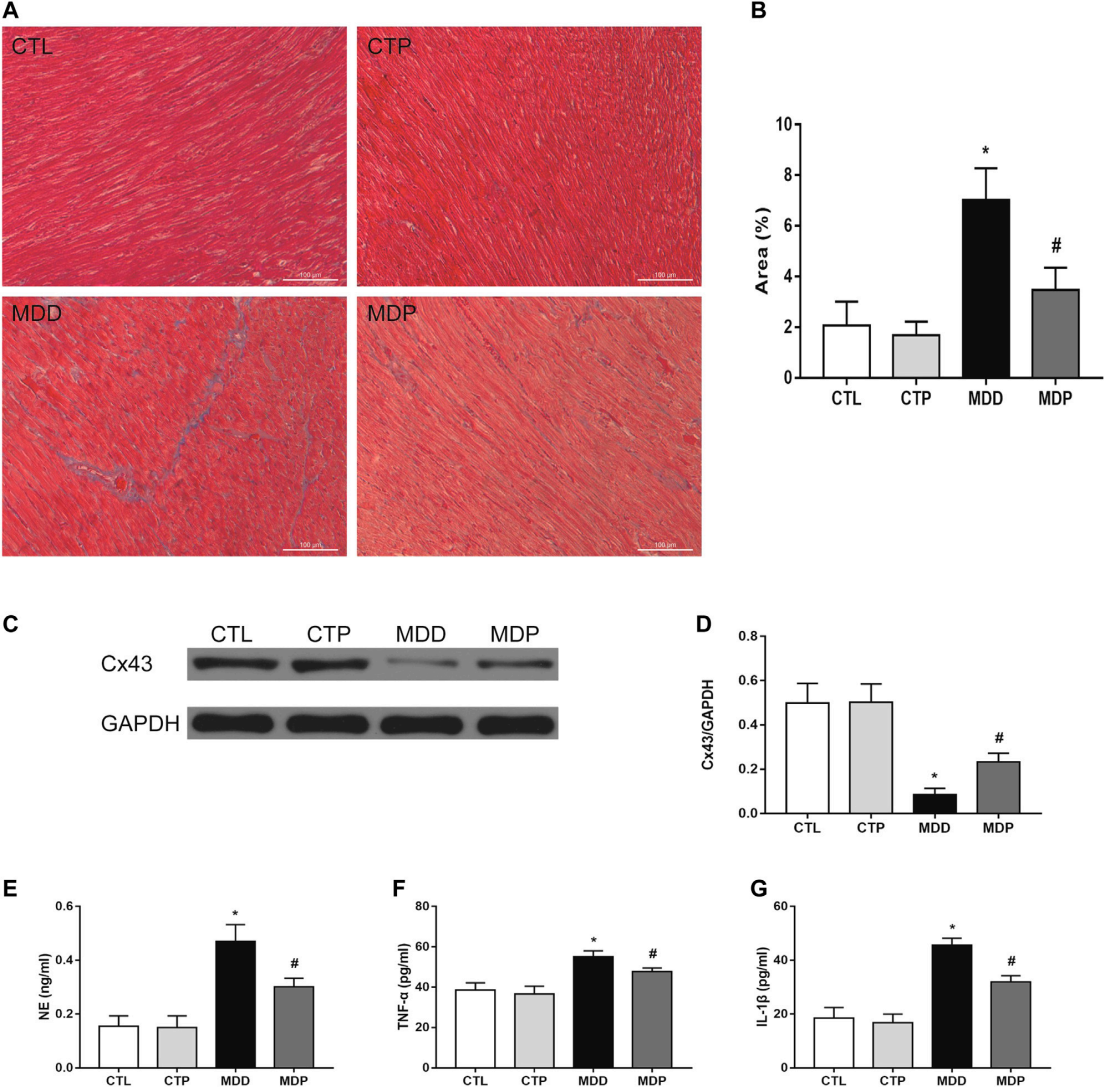
Ventricular myocardial fibrosis, Cx43 expression, and ELISA. **(A,B)** Representative images of Masson staining in LV and quantification of the fibrotic area (original magnification: ×200), respectively. n = 7 per group. **(C,D)** Immunoblotting and expression ratio of Cx43, respectively. n = 7 per group. **(E–G)** Serum NE, TNF-α, and IL-1β concentrations, respectively. n = 7 per group. *p < 0.01 vs. CTL; #*p* < 0.01 vs. MDD. LV, left ventricle.

Figure 8A displayed the representative image of Masson staining. The MDD rats showed a significant increase in the ventricular fibrosis compared with the CTL rats, which was then significantly decreased after pinocembrin treatment (Figure 8B). The Cx43 expression was dramatically reduced in MDD rats compared with the CTL rats (0.09 ± 0.01 vs. 0.50 ± 0.03, *p* < 0.01; Figures 8C,D), which was significantly increased after pinocembrin administration in MDP rats. Furthermore, serum NE, TNF-α, and IL-1β were significantly higher in MDD rats than in CTL rats, whereas those were significantly lowered after administration of pinocembrin in MDP rats (Figures 8E–G). No significant differences were found in the abovementioned variables between the CTL group and the CTP group.

### Pinocembrin Upregulated the Expression of Ion Channels and Reduced the Expression Levels of p-p65 and p-IκBα

**FIGURE 9:**
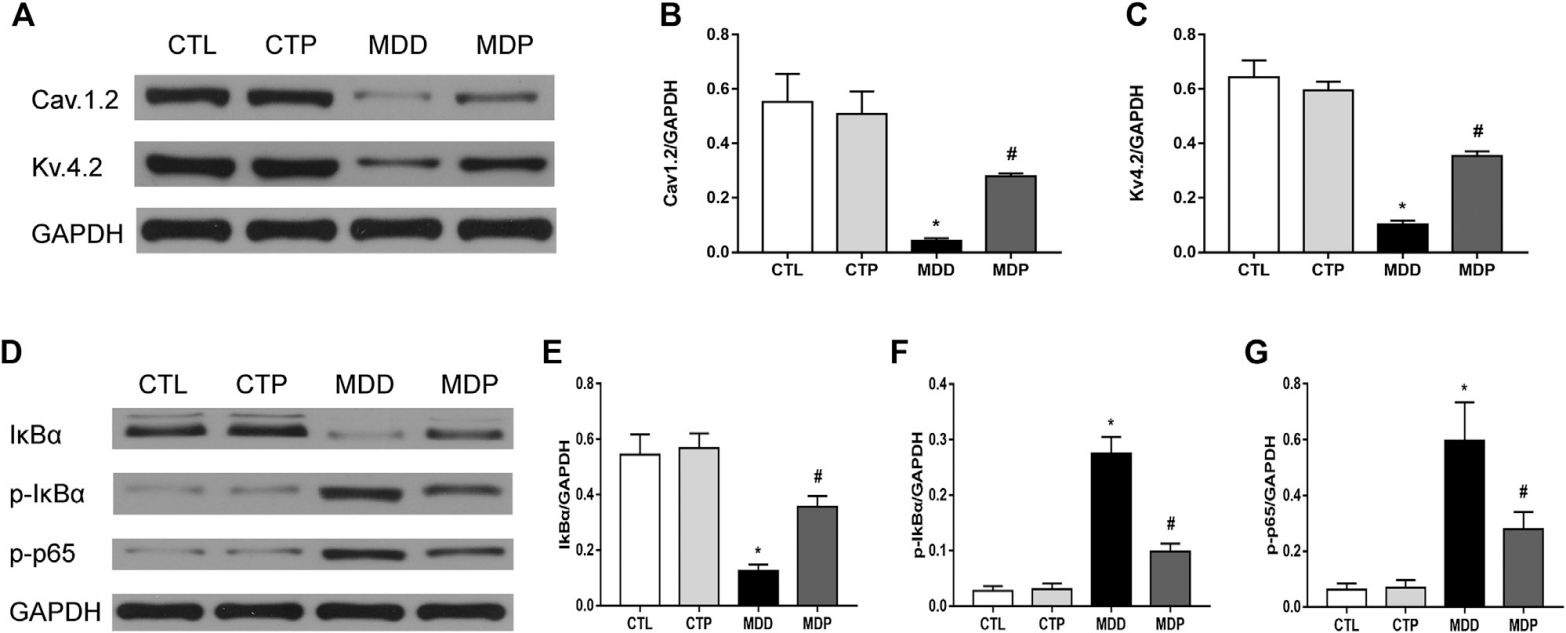
Western blot. **(A–C)** Immunoblotting and expression ratio of Cav1.2 and Kv4.2, respectively. n = 7 per group. **(D–G)** Immunoblotting and expression ratio of IκBα, p-IκBα, and p-p65, respectively. n = 7 per group. *p < 0.01 vs. CTL; #*p* < 0.01 vs. MDD.

As Figures 9A–C showed, compared with the CTL group, the expression of Cav1.2 and Kv4.2 was significantly decreased in the MDD group, whereas it was significantly increased after pinocembrin treatment in the MDP group. A significant reduction in the IκBα expression and increase in the expression of p-p65 and p-IκBα were observed in MDD rats compared with the CTL rats, whereas pinocembrin significantly increased the IκBα expression and reduced the expression levels of p-p65 and p-IκBα in MDP rats (Figures 9D–G). In addition, those showed no significant differences between the CTL group and CTP group.

## Discussion

In this study, we demonstrated that VF incidence was significantly increased in MDD rats. Cardiac autonomic remodeling, ventricular electrical remodeling, ion-channel remodeling, ventricular fibrosis, and enhanced inflammatory responses may serve as the potential indicators, and pinocembrin administration could attenuate the aforementioned parameters, thus decreasing the susceptibility to VF in MDD rats.

Although the transmural dispersion of ventricular ERP and APD was absent, ERP and APD were measured in LV at four epicardial sites. A previous study demonstrated that the ventricular ERP and APD were significantly abbreviated in a canine model of MI, along with the increased risk of VF, whereas prolongation of the ventricular ERP and APD was associated with the decreased susceptibility to VAs (Xiong et al., 2018). The present study showed similar results. Ventricular ERP and APD were significantly abbreviated in the MDD group compared with the CTL group, followed by an increased VF inducibility rate. However, pinocembrin significantly prolonged ERP and APD, with VF inducibility rate decreased in the MDP group, illustrating that pinocembrin ameliorated ventricular electrical remodeling.

QT interval, QTc interval, and Tpeak–Tend interval are markers to predict cardiac arrhythmias, which mainly represent the abnormal repolarization. QTc is a correction of the QT interval, which standardizes the influence of heart rate on QT interval. Although many studies indicate that prolonged QT and QTc interval are associated with VAs, malignant VAs can occur with a shortened QT interval (Dhutia et al., 2016). Actually, VAs may occur at either extreme of the QT interval, evidenced by the long- and short-QT syndromes (LQTS and SQTS). Prolonged Tpeak–Tend often refers to the increased dispersion of repolarization, which is associated with an elevated arrhythmic risk due to the development of the unidirectional block and the subsequent reentry (Opthof et al., 2007). Compared with the CTL group, shortened QT and QTc and prolonged Tpeak–Tend were found in the MDD group in the present study; however, those were significantly alleviated after treatment with pinocembrin, demonstrating the beneficial effects of pinocembrin on modulating the cardiac repolarization.

Cardiac autonomic nervous system (CANS) plays a vital role in the genesis and maintenance of VAs. An imbalance in the CANS, mostly characterized by an augmented sympathetic activity and/or an attenuated parasympathetic activity, may contribute to VAs and even the sudden cardiac death (SCD). Ablation of cardiac sympathetic neurons was found to prolong ERP and APD and decrease VF susceptibility in a canine model of MI (Xiong et al., 2018). Vagus nerve stimulation was also observed to improve ventricular remodeling in MI dogs (Wang et al., 2014). Heart rate variability (HRV), a noninvasive measure of the RR internals, is a useful method to assess the autonomic activity, particularly the parasympathetic system. The time-domain HRV parameters mostly include the SDNN and RMSSD, which often reflect the parasympathetic activity. The increased HR and reduced mean RR also reflect the attenuated parasympathetic activity (Ye et al., 2019a). In this study, the increased HR and reduced mean RR, SDNN, and RMSSD were found in MDD rats, along with the higher NE concentration, indicating that depression led to the augmented sympathetic activity and attenuated parasympathetic activity. The findings also showed that pinocembrin stimulation significantly improved HRV and reduced NE concentration. In addition, the positive nerve densities of TH and GAP43 in LV tissues were increased in MDD rats, along with the increased expression levels of TH and GAP43, confirming the augmented sympathetic control in LV in the MDD group; however, pinocembrin depressed the sympathetic activity. CANS dysfunction was also found in depressed patients compared with the control individuals, evidenced by the higher levels of circulating catecholamines and the decreased HRV, which increased the risk of adverse cardiovascular events, including death (Carney et al., 2001). The results demonstrated that pinocembrin benefited VAs at least in part by regulating the CANS.

The increased ventricular fibrosis and decreased Cx43 expression were detected in MDD rats; however, pinocembrin significantly ameliorated the alterations in MDP rats. Cardiac fibrosis, an important form of cardiac structural remodeling, provides an abnormal substrate of re-entrant arrhythmias, with electrical interferences and heterocellular electrical coupling clarified as the direct mechanisms (Weber et al., 2013). Cardiac fibrosis can promote cardiac hypertrophy and inflammatory responses, which then leads to the increased risk of arrhythmias by promoting cardiac electrical instability (Nguyen et al., 2017). Moreover, tachyarrhythmias may in turn induce myocardial fibrosis, providing a positive feedback to facilitate arrhythmias (He et al., 2011). Gap junctions mediate the ionic current transfer between cardiomyocytes. Alterations of the quantity and spatial distribution of gap junctions are associated with arrhythmogenesis. Cx43 is the most abundant isoform of gap junction proteins in adult hearts. Reduced Cx43 increased the risk of developing ischemia-induced VAs in mice (Lerner et al., 2000). Furthermore, the susceptibility to SCD is significantly increased in Cx43 knockout mice, without deterioration of heart structure and contractile function (Gutstein et al., 2001).

L-type Ca^2+^ channels (ICa-L), located in sarcolemma and activated by membrane depolarization, initiate the excitation-contraction coupling and modulate APD shape in the heart. Cav1.2 is the classical ICa-L which is encoded by CACNA1C or α1. Decreased ICa-L shortens APD, thus modulating the cardiac electrical remodeling (Nattel, 2002). Voltage-gated K^+^ (Kv) channels, in particular the transient outward potassium current (Ito), play an important role in shaping APD and forming the normal cardiac rhythms. Dysregulation of Ito is closely associated with cardiac arrhythmias, including the QT prolongation, early repolarization syndrome, and Brugada syndrome (Johnson et al., 2018). Decreased Ito density was observed in patients with cardiac hypertrophy and heart failure, which was proposed to affect Ca^2+^ currents and lead to AP collapse (Nattel et al., 2007). The expression of Cav.1.2 and Kv.4.2 was reduced in MDD rats in the present study, which was significantly increased after pinocembrin treatment in MDP rats. The results indicated that CUMS contributed to the downregulated ion channels; however, those were upregulated by pinocembrin, thus exhibiting the protective effects on cardiac arrhythmias.

Higher serum TNF-α and IL-1β were observed in MDD rats, showing the activated inflammatory responses in this model. In this study, pinocembrin reduced inflammatory cytokines, illustrating that pinocembrin inhibited MDD-induced inflammatory responses. Clinical studies have demonstrated that inflammatory cytokines were significantly higher in depressed patients than in healthy controls (Whooley and Wong, 2013). A recent study also demonstrated that inflammatory process played an important role in the development of MDD, and inhibiting inflammatory responses could be a potential treatment for improving MDD (Wang et al., 2020). Moreover, previous work indicated that inflammatory conditions could accelerate the occurrence of cardiac arrhythmias (e.g., VAs) in patients (Birnie et al., 2016). The mechanisms by which inflammatory responses contribute to cardiac arrhythmias mainly include the cardiac electrical remodeling and structural remodeling followed by the inflammatory signals. Inflammatory cytokines (e.g. TNF-α and IL-1) may alter the expression of ion channels (e.g. Ca^2+^ and K^+^) (Cutler et al., 2011), thus resulting in APs fluctuations and intracellular Ca2^+^ mishandling. TNF-α can also reduce Cx43 expression (Celes et al., 2007). In addition, inflammation was closely associated with cardiac fibrosis (Shi et al., 2012), thereby providing an abnormal substrate for arrhythmias as mentioned previously. The abovementioned routes can promote the occurrence of cardiac arrhythmias. Nuclear factor-kappa B (NF-κB), an important transcription factor, plays a vital role in regulating inflammatory responses. NF-κB is inactive when binding to inhibitor-κB (IκB) proteins under basic condition, whereas it becomes active after dissociating from IκB under stimuli condition. The activated NF-κB translocates into the nucleus and modulates target genes, such as TNF-α and IL-1β. Previous work indicated that pinocembrin could suppress inflammatory responses by inhibiting the NF-κB signaling pathway (Pei & Sun, 2018). In agreement with our latest study (Ye et al., 2019b), the present results also showed the increased expression of p-p65 and p-IκBα in MDD rats, which was reduced in MDP rats, along with the decreased TNF-α and IL-1β, demonstrating that pinocembrin could suppress inflammatory responses via inhibition of the NF-κB pathway and then decrease the occurrence of cardiac arrhythmias.

## Conclusion

The current study in a rat model of depression indicated that pinocembrin improved autonomic remodeling, ventricular electrical remodeling, ventricular fibrosis, ion-channel remodeling, and inflammatory responses, thereby decreasing VF susceptibility. The results demonstrate that pinocembrin could be a promising strategy for VAs in depressed patients.

## Limitations

First, CUMS is an effective method to induce a rat model of depression according to the previous work (Antoniuk et al., 2019); however, the onset process and physiological changes may differ in human and animal models. Other methods are also expected to create animal models of depression to further prove our present results. Second, although it seems controversial whether the ventricular ERP, APD, and subsequently the cardiac QTc are reduced or prolonged in animals model of depression the previous work has indicated that depression promotes the occurrence of VAs. In the present study, both the ventricular ERP and APD were reduced in MDD rats, along with the increased VF inducibility rate. In fact, the ratio of ERP to APD (e.g. ERP/APD_90_) is more appropriate to predict the risk of cardiac arrhythmias, and the reduced ERP/APD_90_ is more likely to promote cardiac arrhythmias. The index of ERP/APD_90_ should be calculated and the larger sample size in each group should also be employed with the strictly controlled experimental environment in the subsequent study to further confirm the results of the current study. Third, although no major safety concerns were observed after pinocembrin injection in adults in a clinical study (Cao et al., 2015), the VF inducibility was found (1/10) in the CTP group in the present study. Further studies are still needed to prove the effects and safety of pinocembrin on VAs prior to routine clinical use. Fourth, it remains unknown whether the observed electrophysiological effects of pinocembrin are purely as a consequence of the reduction in depressive characteristics, or whether there are any direct effects on the heart. Previous studies have indicated that pinocembrin reduces VAs in a rat model of myocardial I/R by enhancing Na^+^-K^+^ ATPase and Ca^2+^-Mg^2+^ ATPase activity and upregulating Cx43 and Kir2.1 protein expression (Lungkaphin et al., 2015; Zhang et al., 2018). In addition, in our latest study, pinocembrin could ameliorate autonomic remodeling, decreased atrial fibrosis, and reduced inflammatory cytokines, thereby decreasing the occurrence of atrial arrhythmias in rats model of MI (Ye et al., 2019b). The abovementioned studies provide the theoretical feasibility for pinocembrin to ameliorate cardiac arrhythmias at the level of the heart. In the present study, pinocembrin attenuates ventricular electrical remodeling, autonomic remodeling, and ion-channel remodeling (Cav1.2 and Kv4.2), lowers ventricular fibrosis, increases the expression of Cx43, and suppresses the inflammatory responses, which helps to decrease VAs in rats at the level of the heart. Taken together, pinocembrin prevents the susceptibility to VF in CUMS-induced rats not only by ameliorating depression but also by directly acting on the heart. However, further studies are still needed to illustrate the entire mechanisms of VF in depression. Finally, the exposure level and ADME characteristics of pinocembrin in the current study are unknown, which complicates reasoning with effects found in humans. And the matters should be investigated in the subsequent study to provide more evidence on the development of pinocembrin injection.

## Data Availability

The raw data supporting the conclusions of this article will be made available by the authors, without undue reservation.

## References

[B1] Al-Khatib S. M. Arshad A. Balk E. M. Das S. R. Hsu J. C. Joglar J. A. (2016). Risk stratification for arrhythmic events in patients with asymptomatic pre-excitation: a systematic review for the 2015 ACC/AHA/HRS guideline for the management of adult patients with supraventricular tachycardia: a report of the American College of Cardiology/American Heart Association Task Force on Clinical Practice Guidelines and the Heart Rhythm Society. J. Am. Coll. Cardiol. 67 (13), 1624–16 38. 10.1016/j.jacc.2015.09.018 26409260

[B2] Antoniuk S. Bijata M. Ponimaskin E. Wlodarczyk J. (2019). Chronic unpredictable mild stress for modeling depression in rodents: meta-analysis of model reliability. Neurosci. Biobehav. Rev. 99, 101–116. 10.1016/j.neubiorev.2018.12.002 30529362

[B3] Birnie D. H. Nery P. B. Ha A. C. Beanlands R. S. (2016). Cardiac sarcoidosis. J. Am. Coll. Cardiol. 68 (4), 411–421. 10.1016/j.jacc.2016.03.605 27443438

[B4] Cao G. Ying P. Yan B. Xue W. Li K. Shi A. (2015). Pharmacokinetics, safety, and tolerability of single and multiple-doses of pinocembrin injection administered intravenously in healthy subjects. J. Ethnopharmacol. 168, 31–36. 10.1016/j.jep.2015.03.041 25814318

[B5] Carney R. M. Blumenthal J. A. Stein P. K. Watkins L. Catellier D. Berkman L. F. (2001). Depression, heart rate variability, and acute myocardial infarction. Circulation 104 (17), 2024–2028. 10.1161/hc4201.097834 11673340

[B6] Celes M. R. Torres-Duenas D. Alves-Filho J. C. Duarte D. B. Cunha F. Q. Rossi M. A. (2007). Reduction of gap and adherens junction proteins and intercalated disc structural remodeling in the hearts of mice submitted to severe cecal ligation and puncture sepsis. Crit. Care Med. 35 (9), 2176–2185. 10.1097/01.ccm.0000281454.97901.01 17855834

[B7] Chatterjee N. A. Albert C. M. (2019). Sudden arrhythmic death: what is the gold standard? Circ. Arrhythm. Electrophysiol. 12 (7), e007474. 10.1161/CIRCEP.119.007474 31248281 PMC7351044

[B8] Curtis M. J. Hancox J. C. Farkas A. Wainwright C. L. Stables C. L. Saint D. A. (2013). The lambeth conventions (II): guidelines for the study of animal and human ventricular and supraventricular arrhythmias. Pharmacol. Therapeut. 139 (2), 213–248. 10.1016/j.pharmthera.2013.04.008 23588158

[B9] Cutler M. J. Jeyaraj D. Rosenbaum D. S. (2011). Cardiac electrical remodeling in health and disease. Trends Pharmacol. Sci. 32 (3), 174–180. 10.1016/j.tips.2010.12.001 21316769 PMC3073587

[B10] Dhutia H. Malhotra A. Parpia S. Gabus V. Finocchiaro G. Mellor G. (2016). The prevalence and significance of a short QT interval in 18,825 low-risk individuals including athletes. Br. J. Sports Med. 50 (2), 124–129. 10.1136/bjsports-2015-094827 26400956

[B11] Fabbri C. (2016). Genetic and environmental contribution to major depressive disorder and self-declared depression. Ebiomedicine 14, 7–8. 10.1016/j.ebiom.2016.11.030 27916549 PMC5161433

[B12] Grippo A. J. Santos C. M. Johnson R. F. Beltz T. G. Martins J. B. Felder R. B. (2004). Increased susceptibility to ventricular arrhythmias in a rodent model of experimental depression. Am. J. Physiol. Heart Circ. Physiol. 286 (2), H619–H626. 10.1152/ajpheart.00450.2003 14715499

[B13] Gutstein D. E. Morley G. E. Tamaddon H. Vaidya D. Schneider M. D. Chen J. (2001). Conduction slowing and sudden arrhythmic death in mice with cardiac-restricted inactivation of connexin43. Circ. Res. 88 (3), 333–339. 10.1161/01.res.88.3.333 11179202 PMC3630465

[B14] Hammen C. Davila J. Brown G. Ellicott A. Gitlin M. (1992). Psychiatric history and stress: predictors of severity of unipolar depression. J. Abnorm. Psychol. 101 (1), 45–52. 10.1037//0021-843x.101.1.45 1537972

[B15] Hasin D. S. Sarvet A. L. Meyers J. L. Saha T. D. Ruan W. J. Stohl M. (2018). Epidemiology of adult DSM-5 major depressive disorder and its specifiers in the United States. JAMA Psychiatry 75 (4), 336–346. 10.1001/jamapsychiatry.2017.4602 29450462 PMC5875313

[B16] He X. Gao X. Peng L. Wang S. Zhu Y. Ma H. (2011). Atrial fibrillation induces myocardial fibrosis through angiotensin II type 1 receptor-specific Arkadia-mediated downregulation of Smad7. Circ. Res. 108 (2), 164–175. 10.1161/CIRCRESAHA.110.234369 21127293 PMC3035429

[B17] Johnson E. K. Springer S. J. Wang W. Dranoff E. J. Zhang Y. Kanter E. M. (2018). Differential expression and remodeling of transient outward potassium currents in human left ventricles. Circ. Arrhythm. Electrophysiol. 11 (1), e005914. 10.1161/CIRCEP.117.005914 29311162 PMC5775893

[B18] Kilkenny C. Browne W. J. Cuthill I. C. Emerson M. Altman D. G. (2012). Improving bioscience research reporting: the ARRIVE guidelines for reporting animal research. Osteoarthritis Cartilage 20 (4), 256–260. 10.1016/j.joca.2012.02.010 22424462

[B19] Lerner D. L. Yamada K. A. Schuessler R. B. Saffitz J. E. (2000). Accelerated onset and increased incidence of ventricular arrhythmias induced by ischemia in Cx43-deficient mice. Circulation 101 (5), 547–552. 10.1161/01.cir.101.5.547 10662753

[B20] Liang J. Yuan X. Shi S. Wang F. Chen Y. Qu C. (2015). Effect and mechanism of fluoxetine on electrophysiology *in vivo* in a rat model of postmyocardial infarction depression. Drug Des. Dev. Ther. 9, 763–772. 10.2147/DDDT.S75863 PMC4330040 25709400

[B21] Lippi G. Montagnana M. Favaloro E. J. Franchini M. (2009). Mental depression and cardiovascular disease: a multifaceted, bidirectional association. Semin. Thromb. Hemost. 35 (3), 325–336. 10.1055/s-0029-1222611 19452408

[B22] Liu R. Li J. Z. Song J. K. Zhou D. Huang C. Bai X. Y. (2014). Pinocembrin improves cognition and protects the neurovascular unit in Alzheimer related deficits. Neurobiol. Aging 35 (6), 1275–1285. 10.1016/j.neurobiolaging.2013.12.031 24468471

[B23] Liu X. Shi S. Yang H. Qu C. Chen Y. Liang J. (2017). The activation of N-methyl-d-aspartate receptors downregulates transient outward potassium and L-type calcium currents in rat models of depression. Am. J. Physiol. Cell Physiol. 313 (2), C187–C1 96. 10.1152/ajpcell.00092.2017 28566490

[B24] Lungkaphin A. Pongchaidecha A. Palee S. Arjinajarn P. Pompimon W. Chattipakorn N. (2015). Pinocembrin reduces cardiac arrhythmia and infarct size in rats subjected to acute myocardial ischemia/reperfusion. Appl. Physiol. Nutr. Metabol. 40 (10), 1031–1037. 10.1139/apnm-2015-0108 26319563

[B25] Luukinen H. Laippala P. Huikuri H. V. (2003). Depressive symptoms and the risk of sudden cardiac death among the elderly. Eur. Heart J. 24 (22), 2021–2026. 10.1016/j.ehj.2003.09.003 14613738

[B26] Nattel S. (2002). New ideas about atrial fibrillation 50 years on. Nature 415 (6868), 219–226. 10.1038/415219a 11805846

[B27] Nattel S. Maguy A. Le Bouter S. Yeh Y. H. (2007). Arrhythmogenic ion-channel remodeling in the heart: heart failure, myocardial infarction, and atrial fibrillation. Physiol. Rev. 87 (2), 425–456. 10.1152/physrev.00014.2006 17429037

[B28] Nguyen M. N. Kiriazis H. Gao X. M. Du X. J. (2017). Cardiac fibrosis and arrhythmogenesis. Comp. Physiol. 7 (3), 1009–1049. 10.1002/cphy.c160046 28640451

[B29] Opthof T. Coronel R. Wilms-Schopman F. J. Plotnikov A. N. Shlapakova I. N. Danilo P. J. (2007). Dispersion of repolarization in canine ventricle and the electrocardiographic T wave: Tp-e interval does not reflect transmural dispersion. Heart Rhythm 4 (3), 341–348. 10.1016/j.hrthm.2006.11.022 17341400

[B30] Pei B. Sun J. (2018). Pinocembrin alleviates cognition deficits by inhibiting inflammation in diabetic mice. J. Neuroimmunol. 314, 42–49. 10.1016/j.jneuroim.2017.11.006 29150085

[B31] Sapolsky R. M. (1996). Why stress is bad for your brain. Science 273 (5276), 749–750. 10.1126/science.273.5276.749 8701325

[B32] Shen X. Liu Y. Luo X. Yang Z. (2019). Advances in biosynthesis, pharmacology, and pharmacokinetics of pinocembrin, a promising natural small-molecule drug. Molecules 24 (12), 2323. 10.3390/molecules24122323 PMC6631290 31238565

[B33] Shi L. L. Chen B. N. Gao M. Zhang H. A. Li Y. J. Wang L. (2011). The characteristics of therapeutic effect of pinocembrin in transient global brain ischemia/reperfusion rats. Life Sci. 88 (11–12), 521–528. 10.1016/j.lfs.2011.01.011 21262238

[B34] Shi Q. Abusarah J. Baroudi G. Fernandes J. C. Fahmi H. Benderdour M. (2012). Ramipril attenuates lipid peroxidation and cardiac fibrosis in an experimental model of rheumatoid arthritis. Arthritis Res. Ther. 14 (5), R223. 10.1186/ar4062 23079082 PMC3580534

[B35] Shi S. Liu T. Li Y. Qin M. Tang Y. Shen J. Y. (2014). Chronic N-methyl-D-aspartate receptor activation induces cardiac electrical remodeling and increases susceptibility to ventricular arrhythmias. Pacing Clin. Electrophysiol. 37 (10), 1367–1377. 10.1111/pace.12430 24888504

[B36] Tao J. Shen C. Sun Y. Chen W. Yan G. (2018). Neuroprotective effects of pinocembrin on ischemia/reperfusion-induced brain injury by inhibiting autophagy. Biomed. Pharmacother. 106, 1003–1010. 10.1016/j.biopha.2018.07.026 30119165

[B37] Vavakova M. Durackova Z. Trebaticka J. (2015). Markers of oxidative stress and neuroprogression in depression disorder. Oxid. Med. Cell Longev. 2015, 898393. 10.1155/2015/898393 26078821 PMC4453280

[B38] Wang J. Dai M. Cao Q. Yu Q. Luo Q. Shu L. (2019). Carotid baroreceptor stimulation suppresses ventricular fibrillation in canines with chronic heart failure. Basic Res. Cardiol. 114 (6), 41. 10.1007/s00395-019-0750-1 31502080

[B39] Wang X. Zhu L. Hu J. Guo R. Ye S. Liu F. (2020). FGF21 attenuated LPS-induced depressive-like behavior via inhibiting the inflammatory pathway. Front. Pharmacol. 11, 154. 10.3389/fphar.2020.00154 32184729 PMC7058797

[B40] Wang Z. Yu L. Wang S. Huang B. Liao K. Saren G. (2014). Chronic intermittent low-level transcutaneous electrical stimulation of auricular branch of vagus nerve improves left ventricular remodeling in conscious dogs with healed myocardial infarction. Circ. Heart Fail. 7 (6), 1014–1021. 10.1161/CIRCHEARTFAILURE.114.001564 25332149

[B41] Weber K. T. Sun Y. Bhattacharya S. K. Ahokas R. A. Gerling I. C. (2013). Myofibroblast-mediated mechanisms of pathological remodelling of the heart. Nat. Rev. Cardiol. 10 (1), 15–26. 10.1038/nrcardio.2012.158 23207731

[B42] Whooley M. A. Wong J. M. (2013). Depression and cardiovascular disorders. Annu. Rev. Clin. Psychol. 9, 327–354. 10.1146/annurev-clinpsy-050212-185526 23537487

[B43] Xiong L. Liu Y. Zhou M. Wang G. Quan D. Shen C. (2018). Targeted ablation of cardiac sympathetic neurons improves ventricular electrical remodelling in a canine model of chronic myocardial infarction. Europace 20 (12), 2036–2044. 10.1093/europace/euy090 29860489

[B44] Ye T. Liu X. Qu C. Zhang C. Fo Y. Guo Y. (2019a). Chronic inhibition of the sigma-1 receptor exacerbates atrial fibrillation susceptibility in rats by promoting atrial remodeling. Life Sci. 235, 116837. 10.1016/j.lfs.2019.116837 31493481

[B45] Ye T. Zhang C. Wu G. Wan W. Liang J. Liu X. (2019b). Pinocembrin attenuates autonomic dysfunction and atrial fibrillation susceptibility via inhibition of the NF-kappaB/TNF-alpha pathway in a rat model of myocardial infarction. Int. Immunopharm. 77, 105926. 10.1016/j.intimp.2019.105926 31704291

[B46] Zhang P. Xu J. Hu W. Yu D. Bai X. (2018). Effects of pinocembrin pretreatment on connexin 43 (Cx43) protein expression after rat myocardial ischemia-reperfusion and cardiac arrhythmia. Med. Sci. Monit. 24, 5008–5014. 10.12659/MSM.909162 30022020 PMC6063136

