# Corrigendum: Pinocembrin Decreases Ventricular Fibrillation Susceptibility in a Rat Model of Depression

**DOI:** 10.3389/fphar.2021.647320

**Published:** 2021-05-04

**Authors:** Tianxin Ye, Cui Zhang, Gang Wu, Weiguo Wan, Yan Guo, Yuhong Fo, Xiuhuan Chen, Xin Liu, Qian Ran, Jinjun Liang, Shaobo Shi, Bo Yang

**Affiliations:** ^1^ Department of Cardiology, Renmin Hospital of Wuhan University, Wuhan, China; ^2^ Cardiovascular Research Institute, Wuhan University, Wuhan, China; ^3^ Hubei Key Laboratory of Cardiology, Wuhan, China

**Keywords:** depression, ventricular fibrillation, ventricular fibrosis, pinocembrin, autonomic remodeling, ventricular electrical remodeling

In the original article, there was a mistake in Figure 6 as published. The unit of ordinate "s" was incorrectly written as "ms" in Figures 6A-D. The corrected Figure 6 appears below.

**FIGURE 6:**
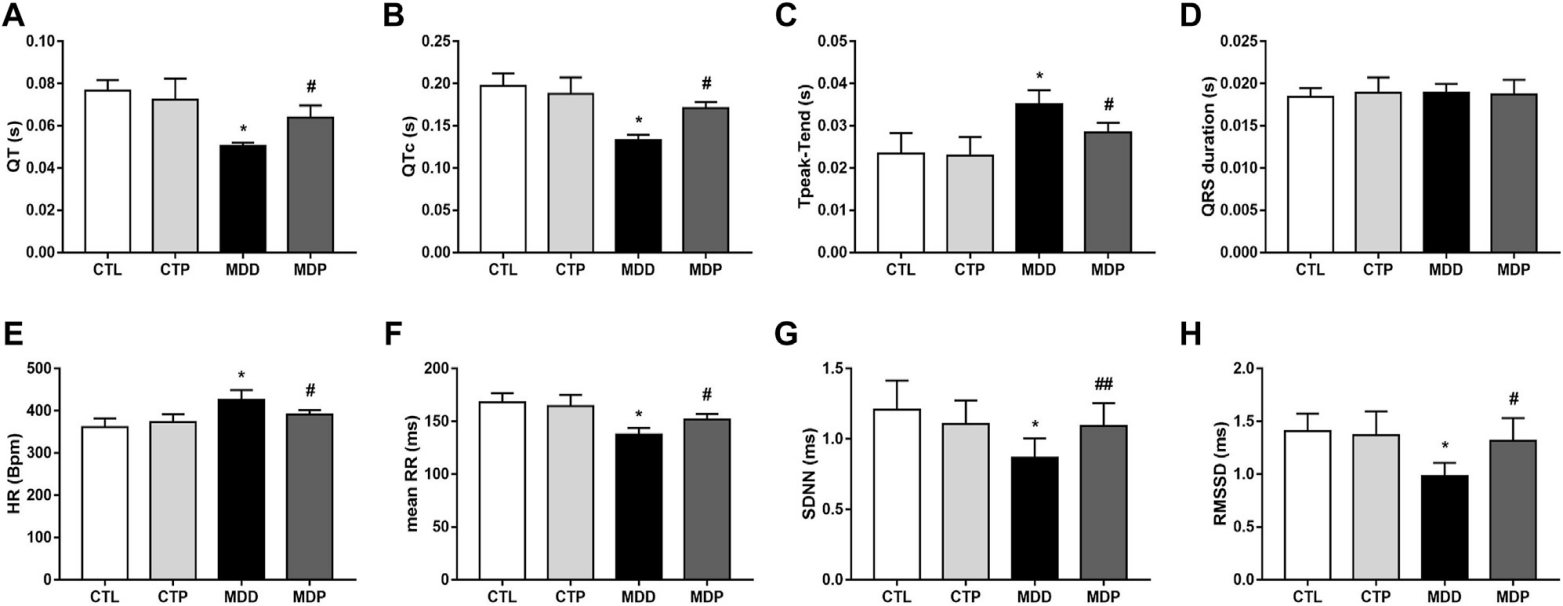
Electrocardiogram parameters and HRV. (A–D) QT interval, corrected QT interval, Tpeak–Tend interval and QRS interval, respectively. n = 7 per group. (E–H) Statistical analysis of HR, mean RR, SDNN, and RMSSD, respectively. n 7 per group. *p < 0.01 vs. CTL; #p < 0.01 vs. MDD; ##p < 0.05 vs. MDD. HRV, heart rate variability.

In the original article, there was an error. We missed the word “increases” in the section of Limitations. A correction has been made to **Limitations,** Sentence 13:

“In the present study, pinocembrin attenuates ventricular electrical remodeling, autonomic remodeling, and ion-channel remodeling (Cav1.2 and Kv4.2), lowers ventricular fibrosis, increases the expression of Cx43, and suppresses the inflammatory responses, which helps to decrease VAs in rats at the level of the heart.”

The authors apologize for this error and state that this does not change the scientific conclusions of the article in any way. The original article has been updated.

